# Supplementary material for: Trends in the Volume and Types of Primary Care Visits during the Two Years of the COVID-19 Pandemic in Israel
Source: Int J Environ Res Public Health. 2022 Aug 25;19(17):10601. doi: 10.3390/ijerph191710601 (PMC9518373; doi:10.3390/ijerph191710601)
Supplement: Supplementary file 1 [file ijerph-19-10601-s001.zip › ijerph-1841717-supplementary.pdf]

Supplementary material:

Figure S1: Daily COVID-19 cases in Israel

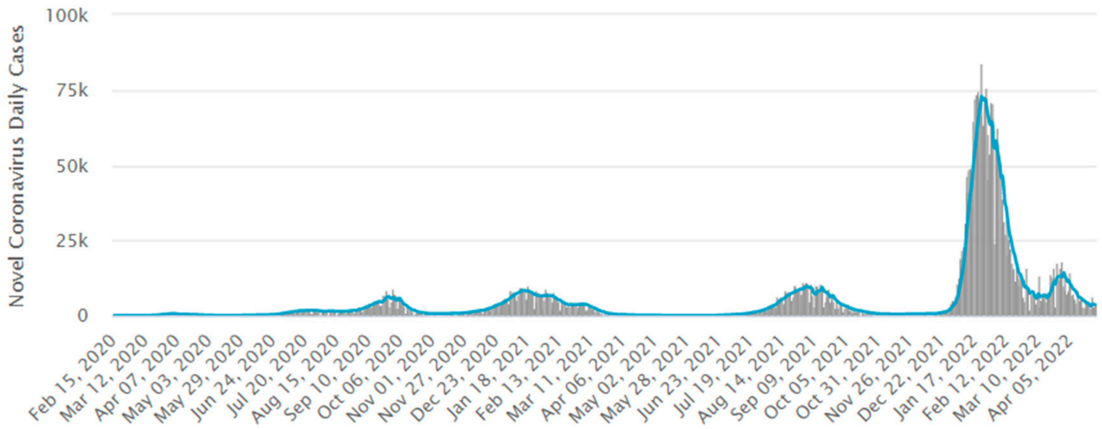

Legend: Y axis indicates Israel's daily numbers of COVID-19 cases. X axis indicates the calendar month.

Source: https://www.worldometers.info/coronavirus/country/israel/

Table S1: Primary care visits by type and period

Type of visit			In-person visits		Phone visits		Asynchronous		Total visits	
Mean daily visit rates per 1000 persons	Pre-COVID year	March–December 2019	11.725		0.678		6.128		18.531	
		January–February 2020	13.21		0.762		7.188		21.16	
	First COVID year	March–December 2020	7.687		3.9		7.856		19.443	
		January–February 2021	7.849		5.285		8.718		21.853	
	Second COVID year	March–December 2021	9.589		3.758		8.353		21.701	
		January–February 2022	9.53		8.68		10.846		29.056	
			Diff	(95% CI)	Diff	(95% CI)	Diff	(95% CI)	Diff	(95% CI)
Differences between corresponding periods	March–December 2019/2020	Diff (95% CI)	-4.038	(-5.436, -2.641)	3.222	(2.618, 3.826)	1.728	(0.702, 2.754)	0.911	(-1.556, 3.379)
		p-value	*0.001>*		*0.001>*		*0.001>*		*NS*	
	January–February 2020/2021	Diff (95% CI)	-5.36	(-5.931, -4.790)	4.523	(-0.120, 9.166)	1.53	(-3.037, 6.098)	0.693	(-8.645, 10.031)
		p-value	*0.001>*		0.053		NS		NS	
	March–December 2019/2021	Diff (95% CI)	-2.135	(-3.533, -0.738)	3.079	(2.475, 3.684)	2.225	(1.199, 3.251)	3.169	(0.702, 5.637)
		p-value	*0.002*		*0.001>*		*0.001>*		*0.01*	
	January–February 2020/2022	Diff (95% CI)	-3.679	(-4.250, -3.109)	7.918	(3.275, 12.561)	3.658	(-0.909, 8.225)	7.897	(-1.441, 17.234)
		p-value	*0.001>*		*0.012*		*0.087*		*0.076*	
	March–December 2020/2021	Diff (95% CI)	1.902	(0.505, 3.301)	-0.143	(-0.747, 0.462)	0.498	(-0.528, 1.524)	2.258	(-0.210, 4.725)
		P-value	*0.006*		*NS*		*NS*		*0.078*	
	January–February 2021/2022	Diff (95% CI)	1.681	(1.110, 2.252)	3.395	(-1.248, 8.038)	2.128	(-2.439, 6.695)	7.204	(-2.134, 16.541)
		P-value	*0.002*		*NS*		*NS*		*0.095*	
